# HUWE1 plays important role in mouse preimplantation embryo development and the dysregulation is associated with poor embryo development in humans

**DOI:** 10.1038/srep37928

**Published:** 2016-11-30

**Authors:** L. J. Chen, W. M. Xu, M. Yang, K. Wang, Y. Chen, X. J. Huang, Q. H. Ma

**Affiliations:** 1 Department of Obstetric and Gynecologic diseases, West China Second University Hospital, Sichuan University, Chengdu 610041, P.R. China; 2 SCU-CUHK Joint Laboratory of Reproductive Medicine, Key Laboratory of Birth Defects and Related Diseases of Women and Children, Ministry of Education, (Sichuan University), West China Second University Hospital, Sichuan University, Chengdu 610041, P.R. China; 3 College of Animal Science & Technology, Nanjing Agriculture University, Nanjing, China

## Abstract

HUWE1 is a HECT domain containing ubiquitin ligase implicated in neurogenesis, spermatogenesis and cancer development. The purpose of the current study is to investigate the role of HUWE1 in early embryo development. Here we demonstrate that *Huwe1* is expressed in both nucleus and cytoplasm of preimplantation mouse embryos as well as gametes. Hypoxia (5% O_2_) treatment could significantly increase *Huwe1* expression during mouse embryo development process. HUWE1 knockdown inhibited normal embryonic development and reduced blastocyst formation, and increased apoptotic cell numbers were observed in the embryos of HUWE1 knockdown group. Human embryo staining result showed that reduced HUWE1 staining was observed in the poor-quality embryos. Furthermore, Western blot result showed that significantly reduced expression of *HUWE1* was observed in the villi of miscarriage embryos compared with the normal control, indicating that reduced expression of *HUWE1* is related to poor embryo development. Oxidative reagent, H_2_O_2_ inhibited *HUWE1* expression in human sperm, indicating that *HUWE1* expression in sperm is regulated by oxidative stress. In conclusion, these results suggest that HUWE1 protein could contribute to preimplantation embryo development and dysregulated expression of *HUWE1* could be related to poor embryo development and miscarriage in IVF clinic.

HUWE1 is a HECT domain containing ubiquitin ligase, which has important roles in neurogenesis, spermatogenesis and cancer development^1^,^2^,^3^. In testis, HUWE1 has been shown as a major histone binding protein with histone ubiquitin activity *in vitro*, indicating that HUWE1 may involve in histone modification during spermatogenesis^1^,^4^. Since *HUWE1* is expressed in the nuclei of spermatogonial stem cells, it has been supposed that HUWE1 may be related to ubiquitination of histones during early meiotic recombination as well as in earlier germ cells and the underlying mechanism is related to hyperactivated DNA damage after *HUWE1* deletion.

The localization of HUWE1 in neuron is similar to that in spermatogonial stem cells as it localized in nucleus in these two cell types, while it localized in the cytoplasm in other somatic cells^1^. The different localization indicates specific substrates of HUWE1 in different cell types. Previous study has demonstrated that HUWE1 could target the anti-apoptotic protein Mcl-1 promoting its ubiquitination and degradation^5^. It has also been shown to ubiquitinate the N-myc transcriptional factor, while such regulation of N-myc appears to be essential for normal differentiation of the cerebral cortex^2^,^6^.

During cancer initiation, HUWE1 has been shown to target p53 by leading its ubiquitination and degradation^3^. p53 is an important transcription factor mediating apoptosis in stress condition such as DNA damage^7^. A recent study has shown that p53 plays a critical role in female reproduction. In the miscarriage patients, the selected haplotype of the p53 is related to the female infertility^8^. Low expression of p53 is also essential for the early development of human embryo, while abnormal activation of p53 could inhibit blastocyst formation and lead to embryo demise^9^.

Whether HUWE1 plays an important role in the development of preimplantation embryo remains unclear. In this study, firstly we investigated the expression and localization of *Huwe1* in mouse embryo, sperm and oocytes, and then studied the role of HUWE1 in embryo development by employing siRNA. Whether poor embryo development is related to diminished HUWE1 expression was also examined in the human embryos collected from IVF clinic. Our results indicate that HUWE1 plays critical roles in apoptosis regulation during preimplantation embryo development.

## Result

### HUWE1 is expressed in preimplantation embryo and gametes

**Figure 1:**
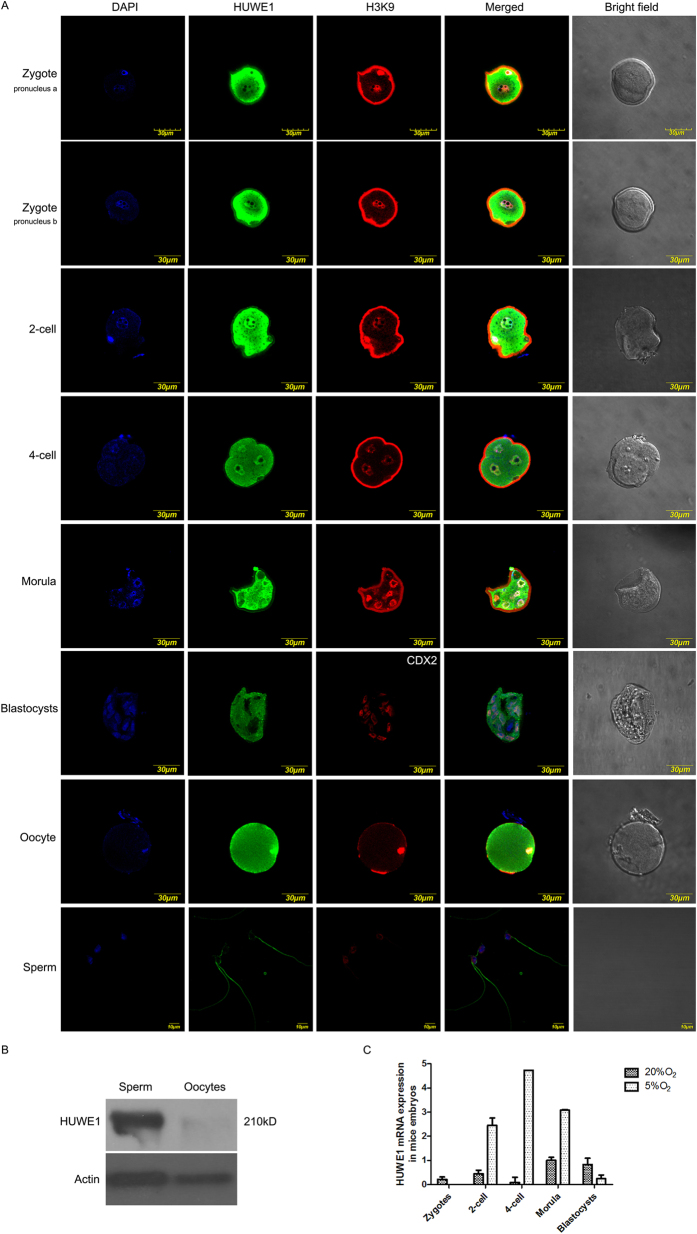
The expression of HUWE1 in mice preimplantation embryos and gametes. The zygotes were obtained 8 h after the beginning of *in vitro* fertilization; the 2-cell embryos, 4-cell embryos, morulae, blastocysts were obtained at the following time points after the zygotes sequentially: 24 h, 48 h, 72 h, 96 h. Scare bar=30 μM. (**A**) Immunofluorescence of HUWE1 and H3K9me2/3 in mice preimplantation embryos and gametes. DAPI was used to stain the nucleus, while in trophoblasts, CDX2 was used instead of H3K9me2/3 to mark the trophectoderm. (**B**) Western blot analysis of HUWE1 in mice sperm and oocytes, β-actin was used as reference gene. The oocytes were deprived of granulosa cells using hyaluronidase to remove somatic cells. (**C**) Quantitative-PCR analysis of *Huwe1* mRNA expression in mice embryos cultured in the atmosphere containing 20% O_2_ and 5% O_2_ respectively. Higher expression of HUWE1 could be detected in the 5% O_2_.

Firstly we checked the expression of mouse *Huwe1* gene in preimplantation embryo development. Immunofluorescence staining result shows that HUWE1 is localized in both nucleus and cytoplasm from zygotes to blastocysts (Fig. 1A). Since H3K9 methylation is a repressive histone modification mark, and is a constitutive heterochromatin marker in embryos, we then used H3K9m2/3 antibody as a marker to manifest heterochromatin of embryo. As well, CDX2 is a trophectoderm marker, which was used here to distinguish the expression patterns of HUWE1 in trophectoderm and inner cell mass respectively. From zygotes to morulae, Huwe1 is expressed in both nucleus and cytoplasm of the early embryos, while in blastocysts, its expression is mainly in trophectoderm. We then checked the localization of HUWE1 in oocytes and sperm, immunofluorescence staining of mouse sperm and oocyte shows that HUWE1 expressed in both nucleus and the cytoplasm of oocytes, while it localized in the whole tail region of mouse sperm. The existence of HUWE1 in mouse sperm was further confirmed by Western blot (Fig. 1B). It has been shown that 5% oxygen in culture could facilitate embryogenesis, and recent study also showed that HUWE1 expression is sensitive to oxidative stress in cancer cell^10^,^11^, we then used 5% oxygen in embryo culture and checked whether the low oxygen could induce *Huwe1* expression in preimplantation embryo. Real time PCR indicates that *Huwe1* gene is expressed in mouse embryos from 2-cell to blastocyst stage with increased expression level. Interestingly, low oxygen could induce *Huwe1* expression from 2-cell to morula stage (Fig. 1C), indicating that HUWE1 expression is regulated by hypoxia condition, such as 5% oxygen treatment.

### HUWE1 protein is important for mouse preimplantation embryo development

**Figure 2:**
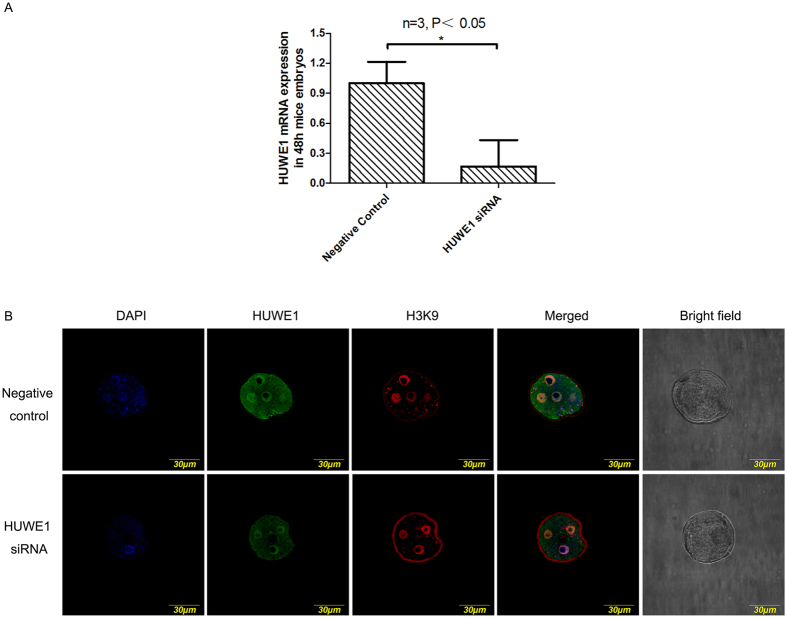
The expression of HUWE1 in mice preimplantation embryos after Huwe1 SiRNA microinjection. The negative control refers to the embryos injected with the control scrambled RNA. (**A**) RT-PCR analysis of *Huwe1*mRNA expression in mice embryos obtained 48 h after microinjection. (**B**) Immunofluorescence of HUWE1 and H3K9 in mice preimplantation embryos obtained 72 h after microinjection. DAPI was used to stain the nucleus. The pictures were captured under the same parameters of staining and confocal setting.

To check whether *Huwe1* gene is important for mouse embryo development, we designed the *Huwe1* siRNA and injected siRNA to zygotes. The efficiency of siRNA was confirmed by the real time PCR in mouse cell line GC2 cell (Supplemental Figure 1). Injection of siRNA caused significant decrease of *Huwe1* expression in embryos compared with control (Fig. 2A). Immunofluorescence staining also confirmed significantly reduced HUWE1 protein expression after 72 hours of injection, confirming that the siRNA could efficiently reduce *Huwe1* expression (Fig. 2B).

**Figure 3:**
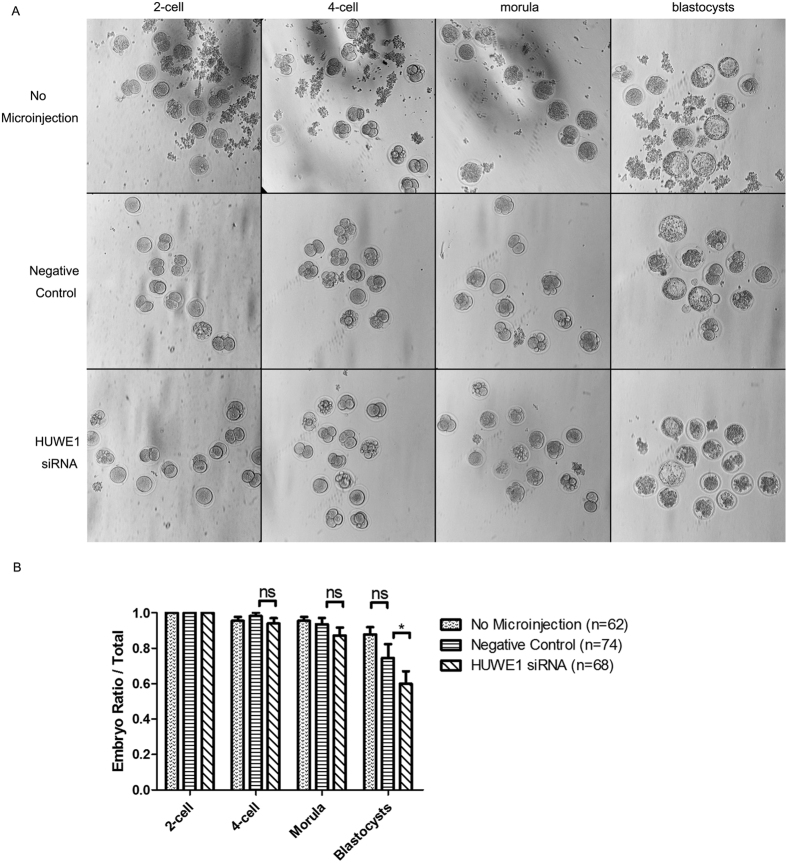
The development of mice preimplantation embryos after *Huwe1* siRNA microinjection. The negative control refers to the embryos injected with the control-siRNA corresponding to *Huwe1*siRNA. No microinjection refers to the embryos without the operation of microinjection. (**A**) Pictures showing different developmental stages of the embryos in culture after different treatments. The objective is 20X. (**B**) Statistical analysis for the developing ratio (the number of embryos that have reached the cell counts required in normal development at each time point over total embryo number) of the embryos after different treatments. *Refers to P < 0.05. ns means no significance in statistics.

**Figure 4:**
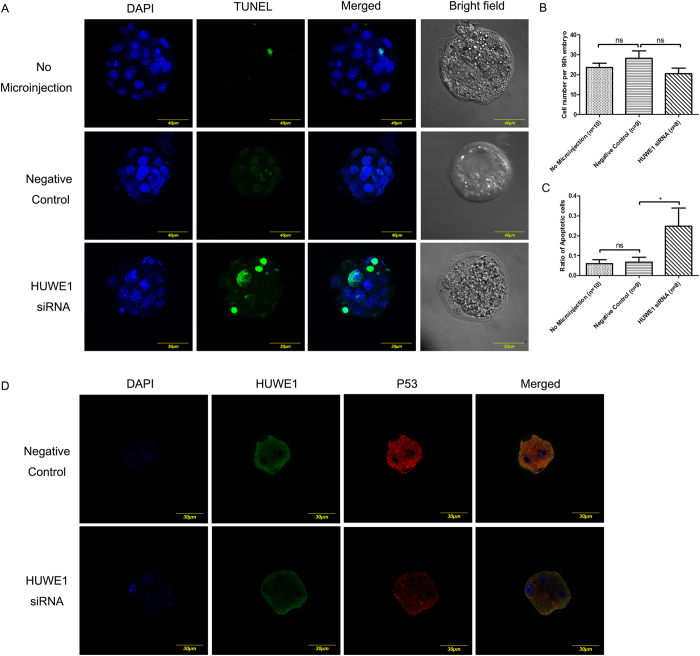
Apoptosis of mice preimplantation embryos after *Huwe1* siRNA microinjection. The negative control refers to the embryos injected with the control RNA corresponding to *Huwe1*siRNA. No microinjection refers to the embryos without the operation of microinjection. (**A**) TUNEL assay for the blastocysts developed from the embryos after different treatments. TUNEL was aimed to show the nuclear substance of the apoptosis cells, while DAPI was used to stain the nuclear substance of all the cells. (**B**) Statistical analysis for the cell counts of each embryos obtained at 96 hours after different treatments. ns means no significance in statistics. (**C**) Statistical analysis for the apoptosis ratio (the number of cells that show positive TUNEL signal over total cell number) of each embryos obtained at 96 hours after different treatments. *Refers to P < 0.05. ns means no significance in statistics. (**D**) Immunofluorescence result showed reduced level of p53 staining in *Huwe1* siRNA injected embryos compared with negative control (scrambled oligos).

To further check whether HUWE1 plays important roles in embryo development, *Huwe1* siRNA was injected and the embryo development was measured at different time points. After injection of Huwe1siRNA, the percentage of blastocysts over total embryos was significantly reduced, indicating that silencing of HUWE1 affect embryo development (Fig. 3). To investigate whether the decreased numbers of blastocysts is caused by apoptosis, we stained the embryo with TUNEL staining and observed the significantly increased number of apoptotic cells after injection of *Huwe1*siRNA, indicating that increased apoptosis is related to the embryo development defect (Fig. 4A–C). Since p53 is a well-known HUWE1 substrate in cancer cell, we then checked whether p53 expression would change after silencing of HUWE1. Unexpectedly, lower expression of p53 was observed after knock down of HUWE1 (Fig. 4D). Therefore, it is unlikely that the apoptosis promoting function of HUWE1 is related to p53. Other substrates may be involved in the phenotype of the *Huwe1* siRNA injected embryos.

### Reduced expression of HUWE1 in villi is related to miscarriage

**Figure 5:**
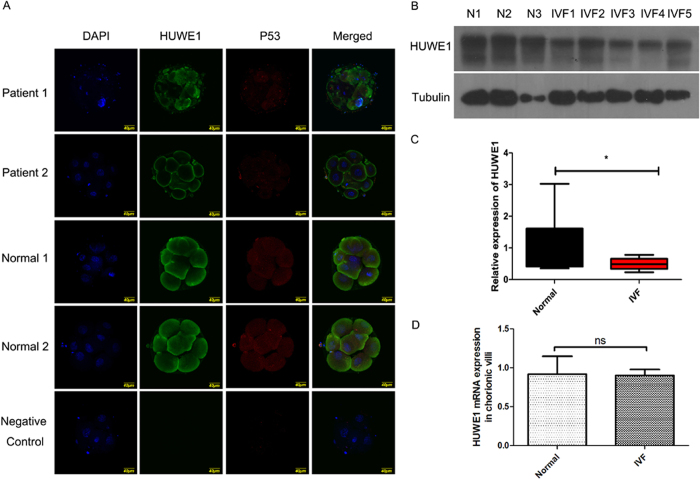
The expression of *HUWE1* in human chorionic villi. The normal refers to the embryos from natural pregnancy and induced abortion, while the IVF refers to the embryos from *in vitro* fertilization but spontaneous miscarriage. The embryos in two groups are of the similar age. (**A**) The staining of HUWE1 and p53 in poor developed embryo (N = 3 for each group). Scare bar = 40 μM. (**B**) Western blot analysis of HUWE1 in human chorionic villi. Tubulin was used as reference gene (N = 14 for control and N = 17 for IVF group). (**C**) Statistical analysis for the relative band intensity (HUWE1 overβ-tubulin) of the western blot showing the reduction of *HUWE1* expression in the embryos from *in vitro* fertilization and spontaneous miscarriage. *Refers to P < 0.05. (**D**) RT-qPCR analysis of *Huwe1* mRNA expression in human chorionic villi. Ns means no significance in statistics (N = 14 for control groups, N = 18 for early miscarriage group).

Poor embryo development is one of the major problems leading to implantation failure in IVF clinic. Since our mouse embryo study shows that Huwe1 knockdown could lead to increased apoptosis in blastocysts, we then determined whether the dysregulated HUWE1 expression is related to poor embryo development in humans. Embryo staining of HUWE1 displayed significantly reduced expression of *HUWE1* in poor-quality embryos (Fig. 5A). We then checked the expressionof *HUWE1* in villi of miscarriage patients. Western blot shows significantly reduced expression of *HUWE1* in the villi samples from miscarriage patients (Fig. 5B,C), further confirming that HUWE1 may play important roles in embryo development. Interestingly, real time PCR shows no significant change of *HUWE1* mRNA levels in the miscarriage patients (Fig. 5D), indicating that the reduced expression of *HUWE1* is possibly caused by post-transcriptional modification instead of transcriptional regulation.

### Oxidative stress inhibits HUWE1 protein expression in human sperm and is possibly related to poor embryo development

**Figure 6:**
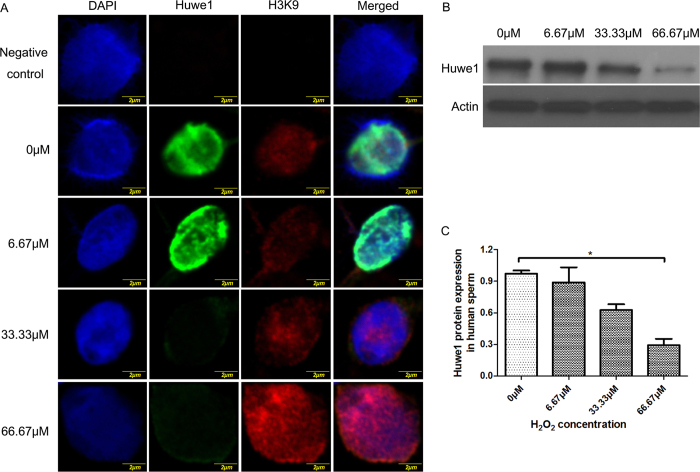
The expression of HUWE1 protein in human sperm after H_2_O_2_ treatment. The fresh semen was washed with PBS and then incubated with H_2_O_2_ of different concentrations at 37 °C for 2 h. (**A**) Immunofluorescence of HUWE1 and H3K9me2/3 in H_2_O_2_ treated human sperm. DAPI was used to stain the nuclear. The negative control refers to human sperm stained without the primary antibody to HUWE1 and H3K9m2/3. (**B**) Western blot analysis of HUWE1 in the H_2_O_2_ treated human sperm. β-actin was used as internal control. (**C**) Statistical analysis for the relative band intensity of the western blot showing the reduction of HUWE1 expression in the H_2_O_2_ treated human sperm. *Refers to P < 0.05. Three independent repeats were analyzed for the data.

Recent study shows that oxidative stress in sperm could have significant impact on the early embryo development^12^, we therefore checked whether the defective embryo development could be related to the expression change of *HUWE1* in sperm. The expression of *HUWE1* in male pronucleus of the tripronuclear cells and the colocalization with paternal heterochromatin marker H3K9me2/3 raise the possibility that paternal chromatin binding HUWE1 protein could play roles in early embryo development (Supplemental Figure 2). We then checked whether human sperm express HUWE1 protein and its expression is regulated by oxidative stress. It has been shown that most histone related protein could be stained only after the decondensation process^13^. Human sperm were treated with *in vitro* decondensation reagent to enlarge the nucleus of the sperm. Immunostaining result shows that HUWE1 could be detected in the chromatin of the sperm, as shown in the colocalization of HUWE1 and H3K9me2/3 (Fig. 6A). To check whether oxidative stress could affect HUWE1 expression, H_2_O_2_ was used to treat sperm and then immunostaining was used to check the expression of the HUWE1 in human sperm. Interestingly, *HUWE1* expression shows dose-dependently decrease in nucleus. Western blot further confirmed a dose-dependent decrease of *HUWE1* expression after oxidative stress, indicating that oxidative stress could reduce the expression of *HUWE1* in nucleus of the sperm (Fig. 6B,C). Together, the human study indicates that HUWE1 in human sperm is regulated by oxidative stress, which could be related to poor embryo development in IVF clinic.

## Discussion

HUWE1 is a HECT domain containing ubiquitin ligase, and recent studies showed that it plays important roles in cancer development, spermatogenesis and stem cell differentiation^1^,^2^,^6^,^14^,^15^. In the current study, our result shows that Huwe1 is expressed in mouse preimplantation embryo, and its expression is regulated by low oxygen concentration during early embryo development. Knockdown of *Huwe1* inhibits the blastocyst formation in mouse, indicating that HUWE1 could play an important role in embryo development. As to the potential target of HUWE1, we show that HUWE1 inhibition does not affect p53 signal. Instead, other targets may mediate the apoptosis promoting function of HUWE1 in the embryos, which needs further investigation.

To explore the translational meaning of the mouse embryo study, we collected the discarded embryos in IVF clinic and villi of miscarriage patients and checked whether the expression of *HUWE1* is related to human embryo development. Our result shows that the expression of *HUWE1* is significantly reduced in poor-quality embryos and villi of miscarriage patients, confirming the important role of HUWE1 in embryo maintenance. Using human sperm as a cell model, our result shows that oxidative stress could inhibit *HUWE1* expression, supporting the notion that oxidative stress regulated HUWE1 protein in sperm could involve in regulation of embryo development. The question comes up as whether the histone modification involves in the effect potentially mediated by HUWE1 on the embryo development?

Recent study shows that paternal methylation of H3K9 plays important roles in intergenerational epigenetic inheritance^13^. Therefore, we stained both Huwe1 and H3K9me2/3 in early embryos and human sperm. Although our result does not support co-localization of Huwe1 and H3K9me2/3 in the morulae and blastocysts stages, H3K9me2/3 and Huwe1 are colocalized in sperm. It is possible that Huwe1 depletion could alter the histone modification, such as the modification of H3K9 in sperm and therefore affect early embryo development. Further mechanistic study could unveil the underlying mechanism for the possible roles of HUWE1 in early embryo development.

Recent data show that oxidative stress in sperm constitutes a major paternal factor determining the early embryo development^12^. Oxidative stress is regarded as an important factor contributing to the male infertility and miscarriage, and the oxidative stress status is affected by environment factors as well as gene mutations during the spermatogenesis^16^,^17^. It has also been shown that DNA damage could induce HUWE1 degradation in cancer cell, which could induce different phenotypes under different stimuli^15^. The exact mechanism of how oxidative stress is involved in HUWE1 regulation is still not clear. However, ubiquitin system has been shown important for sperm function^3^,^18^,^19^. Interestingly, it has been reported that oxidative stress and DNA damage reagent could induce *HUWE1* expression in cancer cell and other system^15^,^20^, therefore, we assume that HUWE1 could be one of oxidative sensitive protein in sperm, that is, the increased DNA damage accelerates the degradation of HUWE1 in sperm. Subsequently, the diminished expression of *HUWE1* affected H3K9 methylation, further compromised embryo development.

It should be noted that although it is likely that sperm born HUWE1 could play critical role in embryo development, oocytes also express HUWE1. Therefore, it is hardly at this stage to distinguish the role of maternal HUWE1 from paternal contribution. Using the sperm of spermatid specific expressed protamine-Cre Huwe1 knockout mouse to do the ICSI and observing the preimplantation embryo development may further confirm the role of paternal HUWE1. Nevertheless, the current study established that HUWE1 could play important roles in early embryo development, at least in blastocyst stage, and sperm born HUWE1 could possibly contribute to the apoptosis regulation from morula to blastocyst development.

It remains to investigate whether HUWE1 could affect the extra-embryonic tissue development. Interestingly, a recent study shows that Cul4b, a Cul domain E3 ubiquitin ligase mediated the degradation of HUWE1^20^. Cul4b, an X-linked mental retardation protein, was also shown to be critical for extra-embryonic tissue development, while dispensable for embryo proper development^21^,^22^. Since both Cul4b and HUWE1 are involved in the X-linked mental retardation, it would be interesting to generate embryo specific and extra-embryonic tissue specific knock out mice and check whether they have neuron specific phenotype^2^,^6^,^14^. It would also be valuable to check the three layers’ development in *Huwe1* knock out embryo. Nevertheless, the detailed follow-up study would unveil the effect of HUWE1 in embryo development as well as the underlying mechanism.

## Materials and Methods

### Experimental animals and Human sample ethics statements

The protocols related to animal operations were checked and approved by the institutional research ethics committee of Sichuan University. All experiments were performed in accordance with guidelines and regulations of West China Second University Hospital of Sichuan University. Using of human tissues was in accordance with ethical guidelines of the West China Second University Hospital. The experiments were approved by the medical ethics committee of West China Second University. Written informed consent was obtained for all subjects. SPF grade ICR mice were bought from Dashuo Laboratory Animal Co. Ltd. (Chengdu, China), and were kept in the experimental animal center of West China Second University Hospital, Sichuan University. All the mice had unlimited access to water and food, and lived in an environment at 25 °C, under 10/14 hours light/dark cycle and the humidity around 70%.

### Mice gametes collecting, *in vitro* fertilization and culture of the early embryos

To induce superovulation, 6-week old female ICR mice were selected, and each of them was treated through intraperitoneal injection with 10IU PMSG (ChiFengBoen Of Inner Mongolia Pharmaceutical Co. Ltd., [2012]050074564) followed by 10IU HCG (Livzon Pharmaceutical Group Co., Ltd. H44020673) 48~50 hours later. Around 16 hours after HCG treatment, the female mice were sacrificed, and the clusters of cumulus-enclosed eggs were collected from their oviducts in HTF medium and kept in incubator with an atmosphere of 5% CO_2_ and 20% O_2_ and temperature at 37 °C. Simultaneously, semen was collected from the caudal epididymis of freshly sacrificed male mice, diluted in HTF medium and incubated in an atmosphere of 5% CO_2_, 20% O_2_, at 37 °C for 1 hour to complete capacitation. Capacitated sperm suspension was added at the concentration of 0.5~1.0 × 10^6^ cell/ml into the HTF medium containing eggs for *in vitro* fertilization which took about 4 hours in an atmosphere of 5% CO_2_ and 20% O_2_, at 37 °C. After fertilization, the assumed fertilized eggs were ready for receiving treatments and culturing as planned.

Mice embryos were cultured in 20 μl droplets of KSOM medium immersed with oil for tissue culture (*In-Vitro* Fertilization, Inc., 4008 P), and incubated at 37 °C in an atmosphere of 5% CO_2_, 20% O_2_ or 5% O_2_ according to the experimental schedule. The developmental status of the embryos were observed through Nikon ECLIPSE TS100 microscope (Nikon, Japan) and photos were taken by iPhone5s (Apple Inc., USA).

### SiRNA oligonucleotides

Three siRNAs targeting mouse *Huwe1* and the negative control were ordered from Guangzhou RiboBio Co. Ltd. After testing their interference efficiency on mouse GC2 cell line by transfection and QPCR, si-m-*Huwe1*_001 was chosen for microinjection. The sequence information of si-m-*Huwe1*_001 (siG141027143417) is as follows: (sense)5′-CCGAGGAACGUAUACCUAUdTdT-3′, (antisense) 3′-dTdTGGCUCCUUGCAUAUGGAUA-5′. The corresponding negative control is a random sequence named as NControl (siN05815122147, Ribobio, Guangzhou, China). The interference efficiency of the chosen *Huwe1* siRNA compared to the negative control were also confirmed on mice embryos by QPCR and immunofluorescence after 48 hours and 72 hours of microinjection respectively.

### Mice zygotes microinjection

Microinjection conducted on the assumed fertilized eggs of mice was done using Narishige NT-88-V3 manipulators and IM-9B microinjector (Narishige Inc., Japan) as well as commercial injection pipettes (JIEYING Laboratory Inc., I-35) with outer diameter as 6.5 μm and inner diameter as 5.5 μm. The volume of fluid injected into each embryo was around 275 fL, and the concentration of *Huwe1* siRNA and its negative control were both 20 μmol/L. The microinjection were repeated more than three times, and over 100 embryos in total were recruited in each treatment group. After operation, embryos were transferred into KSOM medium and cultured in an atmosphere of 5% CO_2_ and 20% O_2_, at 37 °C as described above. They were monitored for specific stages of development and harvested for follow-up detecting assays as planned.

### Cell counting and TUNEL assay

Mice embryos at the 96h-stage of development, which were morphologically compacted morulae or blastocysts, were collected to detect apoptosis between the control and experiment groups. The terminal deoxynucleotidyl transferase dUTP nick end labeling (TUNEL) assay was used by an *In Situ* Cell Death Detection Kit (Roche, 11684795910, USA) according to the manufacturer’s protocol. Briefly, after fixing in 4% PFA then permeabilizing in 0.5% triton X-100, TUNEL reaction mixture was used to label DNA strand breaks, and DAPI was subsequently used to show the nuclei of embryos. The images of embryos were captured in depth by Olympus FLUOVIEW FV1000 laser confocal microscope (Olympus, Japan), and they were used for the cell counting by two individuals who were unware of the group information of treatments.

### Removing zona pellucida and Immunofluorescence of early embryos

Embryos were incubated in Acidified medium (SAGE IVF Inc., 4013) until the Zona Pellucida was removed, the reaction was stopped in culture medium. Then embryos were washed three times for 15 min in 1% BSA/PBS, fixed in 3.7% PFA in PBS for 1 hour, permeabilized with 0.5% Triton-X-100 in PBS for 30 minutes and blocked in 1% BSA/PBS for 1 h at room temperature. Subsequently embryos were incubated in primary antibody (HUWE1 rabbit anti-human polyclonal antibody, LifeSpan BioSciences, Inc., LS-B1359; USA) diluted 1:200 in blocking solution overnight at 4 °C. Other antibodies including p53 (1:100, Zhengnen Biological, Chengdu, China), H3K9m2/3 (1:200, Cell signaling, Beverly, USA), CDX2 (1:100, Zhengnen Biological, Chengdu, China) were also used. Embryos were washed five times in 1% BSA/PBS, followed by incubation of secondary antibody (Life Technologies, USA) diluted 1:500 in blocking solution in dark for 1 h at room temperature. Embryos were washed five times in 1% BSA/PBS, after then incubated in 2 μg/ml DAPI for 8 min, washed once and mounted on glass slides with Gold anti-fade reagent (P36934, Life Technologies, USA). In order to ensure the comparability between embryos of patients and the normal, all samples were stained simultaneously and with double blinded way.

### H_2_O_2_ treatment of human sperm, sperm head decondensation and immunofluorescence

Sperm samples were washed with PBS and then diluted to 1 × 10^7^/ml. 30% H_2_O_2_ were added in the volume of 1 μl, 5 μl and 10 μl to the total of 1.5 ml sample and then were treated for 3 hours before analysis with Western blot.

For immunostaining, 1 ml of sperm suspension was centrifuged at 1,000 rpm for 6 min, the sediment was incubated in 100 μl decondensation buffer (2.5 mM DTT, 0.2% Triton-X-100 in PBS) for 20 min, then incubated in 0.5% (v/v) heparin in decondensation buffer for 4 min. 10 μl of the decondensated sperm suspension was brought onto a glass slide and spread out with the pipet tip, then air dried. The slide was subsequently incubated in 4% PFA for 15 min, air dried, washed in photo-fluo diluted 1:200 in PBS. After drying, the slide was washed in PBS-T twice, permeabilized with 0.2% Triton-X-100 in PBS for 15 min and blocked in 5% BSA/PBS for 1 h at room temperature, followed by incubation of primary antibody diluted 1:200 in blocking solution overnight at 4 °C. The slide was washed in PBS-T three times for 15 min, then incubated in secondary antibody diluted 1:500 in blocking solution in dark for 1 h at room temperature and washed three times in PBS-T. After then the slide was incubated in 2 μg/ml DAPI for 8 min, washed once and mounted with Gold anti-fade reagent (Life Technologies).

### QPCR

The Single Cell-to-CT™ Kit (Cat:4458237, Life Technology, USA) workflow is comprised of 4 functional steps: cell lysis, reverse transcription, cDNA pre-amplification and Real-Time PCR as outlined in the Kit instruction, 6–10 embryos were used for each group and Taqman based method was used to check Huwe1 expression (Taman probe: mHUWE1, Mm00315533, Life Technology, USA). 18 S probe was used as internal control in the real time result. ABI 7500 real time PCR system was used for amplification.

For villi samples, SYBR green based method was used. The primers’ sequences of hHUWE1 are as follows: Forward primer: CAAACTACATCACTCGTCTGGG; Reverse primer: AGTCTCTGCAACATTCTGCAAG. 500 ng of RNA was used for reverse transcription. The relative mRNA expression levels were calculated using the 2−ΔΔCT method, where ΔCT refers to the subtraction of the CT of Actin or U6 from the mRNA, and ΔΔCT was calculated by subtracting the ΔCT.

### Western Blot

The whole cells lysates were prepared with RIPA cell lysis buffer with addition of DMSF and proteinase inhibitors. Equal amount of proteins were resolved by 10% SDS-PAGE and then transferred to PVDF membrane. The membranes were incubated with a primary HUWE1 antibody at the concentration of 1:500 (LS-B1359, Lifespan, USA) and normalized to β-actin control at the concentration of 1:5000 (20130822, Zen Bioscience, China). The primary antibody binding was visualized with horseradish peroxidase-conjugated goat anti-rabbit or anti-mouse IgG (1:10000, ZSGB-BIO, China). The bind intensities were measured using HRP (1305702, Millipore Corporation, Billerica, USA) and image analysis software.

### Clinical procedure and embryo evaluation

Using of discarded human embryos was approved by the Institutional Ethics Committee of West China Second University Hospital of Sichuan University. All patients involved in human embryo immunostaining experiment experienced GnRH agonist long-term stimulation protocol or GnRH agonist short-term protocol, depending on ovarian reserve, assessed by the patient’s age, baseline serum FSH concentration, previous ovarian response to gonadotropins, and the preference of each clinician. Oocyte retrieval was scheduled 36–38 hours later guided by transvaginal ultrasound. 40 hours later after HCG triggering, standard IVF or ICSI procedures, depending on semen parameters or previous laboratory results, were used to achieve oocytefertilization and embryos for transfer.

On day 3, all embryos were graded on a scale of 0 to 4, which was based on a modification of Veeck’s morphological grading system. 1 to 3 embryos transfer was performed 3 days after oocyte retrieval. After transfer, poor quality embryos (Veeck’s score was equal to III or IV) were sent to biologic laboratory for detecting expression of HUWE1.

### Chorionic villi tissues

Chorionic villi tissues were extracted from patients who were proved to be miscarriages after clinical pregnancy during their IVF procedure. Control normal chorionic villi were taken from women who underwent artificial abortion procedure because of unplanned pregnancy. All these patients did not have known anatomic or genetic abnormalities, and all villa tissues were proved with normal chromosome karyotype and the gestional age for every sample was shown in Supplemental Table 1.

### Statistical Analysis

The experimental data analysis was performed with GraphPad Prism 5 software. Data were presented as means ± SEM in the figure. T test was used for two-sample test of homogeneity of variance between groups. For three or more groups, one-way ANOVA and Newman-Keulspost test was used for the comparison. P < 0.05 was considered statistically significant.

## Additional Information

**How to cite this article**: Chen, L. J. *et al*. HUWE1 plays important role in mouse preimplantation embryo development and the dysregulation is associated with poor embryo development in humans. *Sci. Rep.* **6**, 37928; doi: 10.1038/srep37928 (2016).

**Publisher's note:** Springer Nature remains neutral with regard to jurisdictional claims in published maps and institutional affiliations.

## Supplementary Material

Supplementary Information

Supplementary Dataset
