# Supplementary material for: HUWE1 plays important role in mouse preimplantation embryo development and the dysregulation is associated with poor embryo development in humans
Source: Sci Rep. 2016 Nov 30;6:37928. doi: 10.1038/srep37928 (PMC5128802; doi:10.1038/srep37928)

Supplemental information

Title:

HUWE1 play important role in mouse preimplantation embryo development and the dysregulation is associated with poor embryo development in humans

L.J. Chen， W. M. Xu, M. Yang, K.Wang, Y. Chen, Xianju Huang, Q. H. Ma

Figure 1 Legend: The relative expression of Huwe1 after transfection of different concentration of Huwe1 siRNA.

Figure 2 Legend: immunostaining result of HUWE1 in tripronuclear zygote

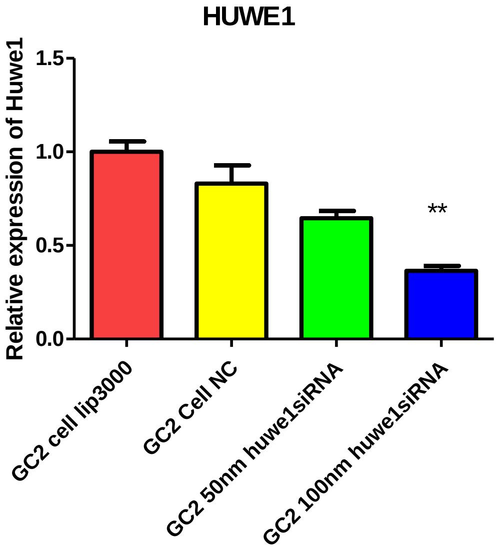

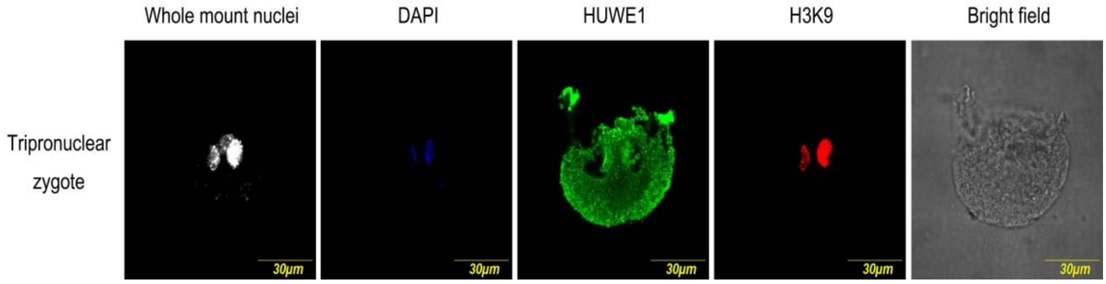

Supplement: Supplementary Information [file srep37928-s1.pdf]
